# Giant Trigeminal Schwannoma Presenting with Obstructive Hydrocephalus

**DOI:** 10.7759/cureus.386

**Published:** 2015-11-20

**Authors:** Ignacio Jusué-Torres, Juan Carlos Martinez-Gutierrez, Benjamin D Elder, Alessandro Olivi

**Affiliations:** 1 Department of Neurosurgery, Johns Hopkins University School of Medicine

**Keywords:** trigeminal schwannoma, obstructive hydrocephalus, natural history, neurosurgery, retro-sigmoid craniotomy

## Abstract

Trigeminal schwannomas represent between 0.07% and 0.36% of all intracranial tumors and 0.8% to 8% of intracranial schwannomas. Selection of the appropriate management strategy requires an understanding of the tumor’s natural history and treatment outcomes. This report describes the case of a 36-year-old male who presented with a three-month history of progressive headaches, dizziness, loss of balance, decreased sleep, and cognitive decline. Magnetic resonance imaging revealed a large enhancing lesion centered around the left Meckel’s cave and extending into both the middle and the posterior fossa with obstructive hydrocephalus secondary to compression of the fourth ventricle. Resection of the posterior fossa component of the tumor was performed in order to relieve the mass effect upon the brainstem without attempting a radical removal of the middle fossa component and a potential risk of further cognitive impairment. The pathological exam confirmed the diagnosis of a trigeminal schwannoma. The residual tumor showed progressive spontaneous volumetric shrinkage after a subtotal surgical resection. This case shows the value of a planned conservative surgery in complex schwannomas and highlights the challenges in interpreting the treatment responses in these benign tumors, whether approached surgically or with stereotactic radiation techniques.

## Introduction

According to the most recent data from the Central Brain Tumor Registry of the United States (CBTRUS) [1], schwannomas account for 8% of all primary brain and central nervous system tumors. They are the third most common histology found in the 20 to 44-year-old age range. The incidence of schwannomas is 1.57 per 100,000, with the third highest incidence for non-malignant tumors after meningiomas (7.33 per 100,000) and pituitary tumors (3.12 per 100,000). However, schwannomas arising from the intracranial portion of the trigeminal nerve are rare. They represent only 0.07%-0.36% of all intracranial tumors and 0.8% to 8% of intracranial schwannomas [2].

The management of trigeminal schwannomas generally includes the following options: observation, microsurgical resection, radiosurgery, fractionated conformal radiotherapy, or a combination approach with surgery and radiotherapy. Selection of the appropriate management strategy is based on a detailed understanding of the natural history and treatment outcomes, as well as on the specific clinical presentation. The presented clinical case provides an example of a challenging clinical situation where all the relevant factors (natural history, presenting symptoms, and anatomical and functional risks) were considered in order to optimize the treatment.

## Case presentation

A 36-year-old previously healthy male presented with a three-month history of progressive headaches, dizziness, loss of balance, decreased sleep, and cognitive impairment with psychosocial problems. On physical exam, the patient had normal mental status but had difficulty with tandem gait, truncal ataxia, and mild left sixth nerve palsy with diplopia. There was mildly decreased sensation on the left face in all three branches of the trigeminal nerve with an intact corneal reflex. Informed patient consent was obtained.

**Figure 1:**
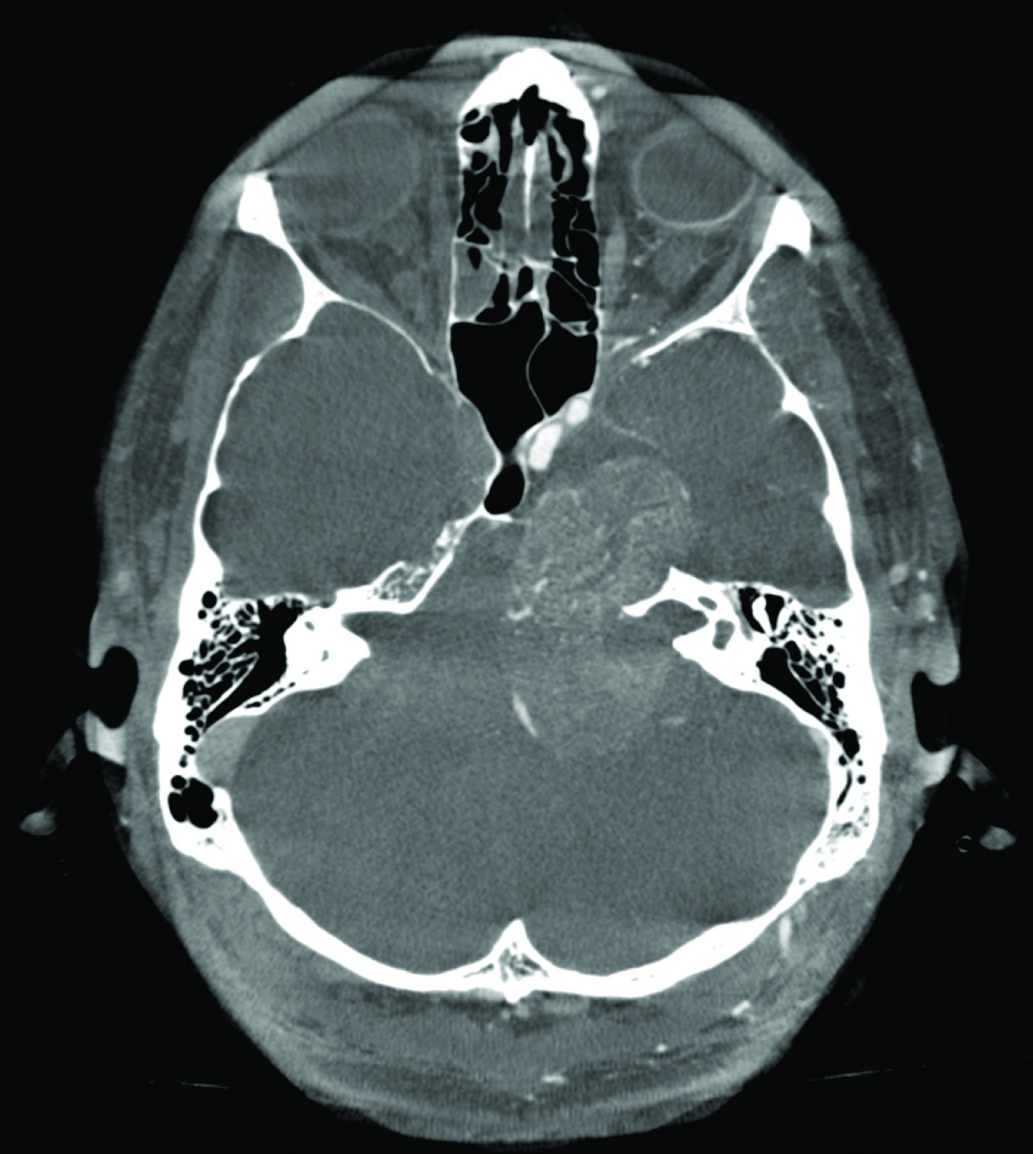
Preoperative CT scan Axial computed tomography (CT) scan (bone windows) showing a lesion at the level of the left Meckel´s cave with significant petrous bone remodeling.

On an initial computed tomography (CT) scan, a middle and posterior fossa mass was noted with a significant remodeling of the petrous bone (Figure 1).

**Figure 2:**
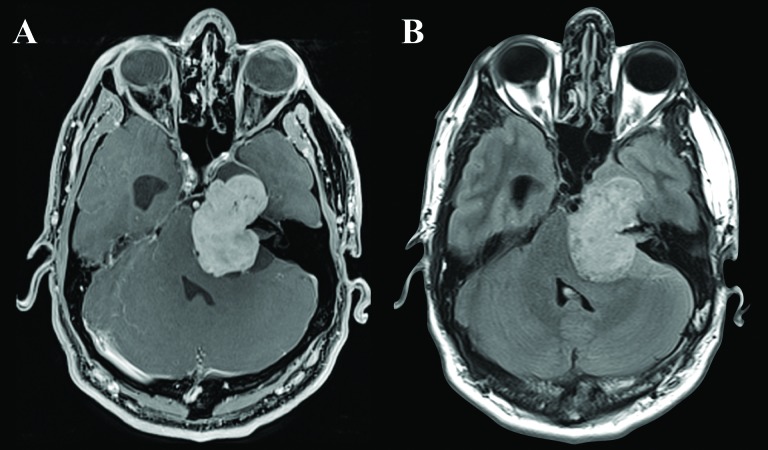
Preoperative MRI A. Axial T1-weighted magnetic resonance imaging (MRI) with gadolinium showing a homogenously enhancing extra-axial lesion, with significant mass effect on the brain stem, fourth ventricle, and left temporal lobe, with an enlarged right ventricular temporal horn. B. Axial fluid-attenuated inversion recovery (FLAIR) weighted MRI showing lack of edema in brainstem, cerebellum and left temporal lobe.

Magnetic resonance imaging (MRI) revealed a contrast-enhancing T1 hypointense and a fluid-attenuated inversion recovery (FLAIR) hyperintense mass in the left middle and posterior fossa (Figure 2).

**Figure 3:**
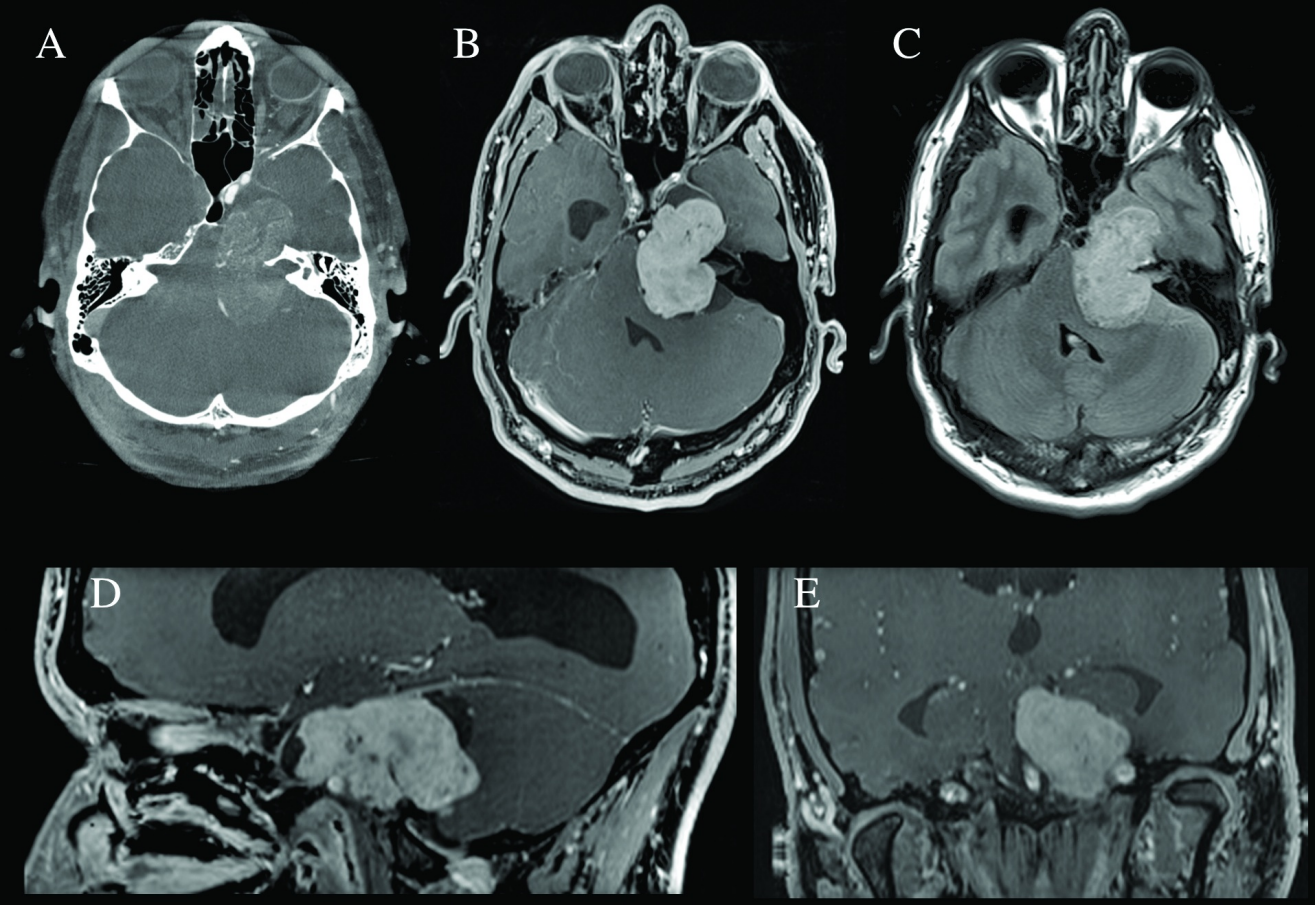
Preoperative imaging A. Axial computed tomography (CT) scan (bone windows) showing a lesion at the level of the left Meckel´s cave with significant petrous bone remodeling. B. Axial T1-weighted magnetic resonance imaging (MRI) with gadolinium showing a homogenously enhancing extra-axial lesion, with significant mass effect on the brain stem, fourth ventricle, and left temporal lobe, with an enlarged right ventricular temporal horn. C. Axial fluid-attenuated inversion recovery (FLAIR) weighted MRI showing lack of edema in brainstem, cerebellum and left temporal lobe. D. Sagittal T1-weighted MRI with gadolinium showing the tumor´s middle and posterior fossa involvement as well as the anatomical relationship with the petrosal portion of the left carotid artery and the enlarged lateral ventricle. E. Coronal section demonstrating an enlarged supratentorial ventricular system and tumor adjacent to the bilateral carotid arteries.

The extra-axial lesion extended into the left cavernous sinus from Meckel’s cave, along the course of the trigeminal nerve. The mass measured approximately 4.3 x 3.1 x 4.5 cm (39.50 cc), and exerted a significant mass effect on the pons and fourth ventricle with subsequent obstructive hydrocephalus. A mild mass effect was also present on the mesial temporal lobe (Figure 3).

**Figure 4:**
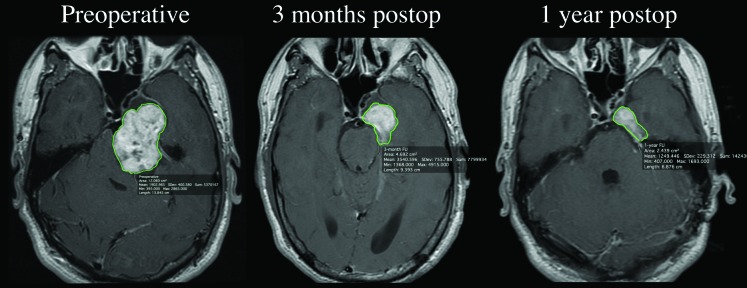
Postoperative imaging Representative axial T-1 weighted post-gadolinium images used for volumetric analysis showing the progressive postsurgical reduction of tumor volume: A. Preoperative; B. Three months postoperatively; C. One-year post-operatively.

With symptoms of hydrocephalus secondary to mass effect from this large neoplastic lesion, microsurgical treatment with excision of the large posterior fossa component of the tumor was recommended. The patient underwent an uncomplicated retrosigmoid craniotomy with resection of the posterior fossa portion of the mass and a subtotal resection of the middle fossa component. Pathology confirmed the diagnosis of trigeminal schwannoma. Three-month follow-up imaging revealed a reduction of the obstructive hydrocephalus and a residual lesion (12.27 cc) that had further regressed 69.7% (3.72 cc) at his one-year follow-up (Figure 4).

The patient reported transient facial numbness postoperatively but had a steady improvement of cognitive function with a return to baseline function within three months.

## Discussion

Given their slow growth, trigeminal schwannomas frequently present with a gradual onset of symptoms with cranial nerve involvement (trigeminal hypesthesia, facial pain, hypoacusis) and headaches [3]. However, symptoms related to the development of obstructive hydrocephalus are a rare initial presentation, and to the best of our knowledge, this case report is the first to highlight this presentation. In this case, the patient’s young age, tumor size (> 30 mm), and significant mass effect on the brainstem with subsequent obstructive hydrocephalus precluded the options of radiosurgery or close observation [4].

A microsurgical middle fossa approach for lesions of this nature is often advocated [5-8]. However, in this case, the severity of the brainstem compression with hydrocephalus and the possibility of causing further deterioration with manipulation of the dominant temporal lobe led to the selection of a retrosigmoid posterior fossa approach. In this case, a relatively conservative approach yielded a subtotal resection but allowed for the relief of the brainstem compression and resolution of the hydrocephalus with the restoration of the patient’s baseline cognition. Furthermore, the residual lesion in the middle fossa underwent spontaneous involution with detectable volume reduction during follow-up. Therefore, additional treatments, such as stereotactic radiosurgery or a separate middle fossa approach, have not been pursued at this time. We believe that the primary objectives of surgical treatment (i.e. definitive pathological diagnosis, brainstem decompression, resolution of hydrocephalus, and restoration of cognitive function) were amply achieved with only a posterior fossa approach.

The exact natural history of schwannomas is unclear, as untreated tumors may grow in some instances, yet may remain dormant for many years and even regress in other cases. Around 6-26% of patients show spontaneous regression of acoustic schwannomas without therapy [6, 8]; however, the postoperative natural history of residual trigeminal schwannomas is much less defined. Some studies reported that subtotal removal is associated with a high rate of tumor recurrence [9] while others reported no evidence of recurrence after subtotal surgical resection [10]. However, only a few cases of regression after surgery have been reported [9]. This report represents a case of spontaneous regression of a trigeminal schwannoma after subtotal resection. The mechanism of this spontaneous postsurgical neoplastic involution is unknown; nevertheless, we hypothesize that a partial devascularization of the lesion and subsequent ischemic involution could have led to such a favorable phenomenon.

## Conclusions

To the best of our knowledge, this is the first reported case of a trigeminal schwannoma presenting with obstructive hydrocephalus. This case illustrates that a limited surgical approach, focusing on decompression of tumor mass effect and relief of obstructive hydrocephalus, is of value in the treatment of a complicated schwannoma and that some tumor regression may occur postoperatively. As such, favorable outcomes can be associated with careful planning and execution of specific goal-oriented surgical approaches.
